# Functional traits drive the contribution of solar radiation to leaf litter decomposition among multiple arid-zone species

**DOI:** 10.1038/srep13217

**Published:** 2015-08-18

**Authors:** Xu Pan, Yao-Bin Song, Guo-Fang Liu, Yu-Kun Hu, Xue-Hua Ye, William K. Cornwell, Andreas Prinzing, Ming Dong, Johannes H.C. Cornelissen

**Affiliations:** 1 Key Laboratory of Hangzhou City for Ecosystem Protection and Restoration, College of Life and Environmental Sciences, Hangzhou Normal University, Hangzhou, China; 2 Institute of Wetland Research, Chinese Academy of Forestry, Beijing, China; 3 State Key Laboratory of Vegetation and Environmental Change, Institute of Botany, Chinese Academy of Sciences, Beijing, China; 4 Department of Ecological Science, VU University, Amsterdam, the Netherlands; 5 Evolution & Ecology Research Centre, School of Biological, Earth and Environmental Sciences, University of New South Wales, Sydney, Australia; 6 Université de Rennes 1, Centre National de la Recherche Scientifique, Rennes, France

## Abstract

In arid zones, strong solar radiation has important consequences for ecosystem processes. To better understand carbon and nutrient dynamics, it is important to know the contribution of solar radiation to leaf litter decomposition of different arid-zone species. Here we investigated: (1) whether such contribution varies among plant species at given irradiance regime, (2) whether interspecific variation in such contribution correlates with interspecific variation in the decomposition rate under shade; and (3) whether this correlation can be explained by leaf traits. We conducted a factorial experiment to determine the effects of solar radiation and environmental moisture for the mass loss and the decomposition constant *k*-values of 13 species litters collected in Northern China. The contribution of solar radiation to leaf litter decomposition varied significantly among species. Solar radiation accelerated decomposition in particular in the species that already decompose quickly under shade. Functional traits, notably specific leaf area, might predict the interspecific variation in that contribution. Our results provide the first empirical evidence for how the effect of solar radiation on decomposition varies among multiple species. Thus, the effect of solar radiation on the carbon flux between biosphere and atmosphere may depend on the species composition of the vegetation.

Leaf litter decomposition is a key carbon and nutrient mobilizing process in ecosystems^1^,^2^,^3^. Variation in leaf litter decomposition rates depends on the climate zone and type of ecosystems, due to variation in (1) abiotic factors, e.g., temperature, moisture and ultraviolet radiation (UV), and (2) biotic factors such as litter quality and composition of decomposer organisms^4^,^5^. There is great interspecific variation in leaf litter decomposition rates, mostly driven by strong “afterlife” effects of species functional traits such as leaf N concentration, lignin concentration, leaf pH and specific leaf area^6^,^7^. Recent meta-analysis results also emphasized that the species contribution to variation in litter decomposition rates within climate zones and ecosystems was even much larger than that driven by climate across biomes^7^. However, interspecific variation in leaf litter decomposition rates has seldom been examined in arid and semi-arid ecosystems, in which solar radiation has a great contribution to decomposition processes^8^,^9^. Therefore, it is important to know whether solar radiation in arid and semi-arid regions can also be an important contributor to interspecific variation in leaf litter decomposition rates relative to interspecific variation in microbially-driven decomposition without strong solar radiation.

Recent research has highlighted the contribution of solar radiation, notably via photodegradation, as a driver of litter turnover in arid and semi-arid ecosystems^8^,^9^,^10^,^11^. Persistent standing dead organic matter, strong solar radiation and low precipitation in dry ecosystems increase the contribution of solar radiation to litter decomposition compared to that in humid, more-shady ecosystems^5^. Solar radiation can make the litter more degradable^11^, possibly via breaking up the organic compounds in plant litter into smaller ones^12^. These smaller compounds are then more susceptible to both dissolved organic C leaching and microbial decay^13^. There is some evidence that solar radiation can change the lignin dynamics of plant litters^14^,^15^, making them susceptible to the enzymes produced by fungi and bacteria. Moreover, solar radiation may interact with other abiotic or biotic factors and the effect of solar radiation might depend on other climatic factors, such as moisture availability^16^,^17^. Also, solar radiation and climate (change) may have an interactive effect on biogeochemical cycling by influencing the chemistry of dead plant materials falling to the soil^17^. However, empirical data to quantify such interactions are virtually absent. Given the unique contribution of solar radiation to the leaf litter decomposition process via photodegradation^9^, it is important to know whether, and to which extent, interspecific variation in the contribution of solar radiation to leaf litter decomposition rate correlates with interspecific variation in the decomposition rate without solar radiation.

Decomposition depends both on climate and the legacy of plant functional traits as litter quality^4^,^6^,^7^,^18^. It is well known that physico-chemical features of leaf litter cause marked interspecific differences in microbially-driven decomposition^4^,^6^,^19^,^20^,^21^. However, we know virtually nothing about possible interactions between litter quality of different plant species and the effects of solar radiation on their decomposition. While we know that traits of different species clearly have a large effect on litter decomposition, it has never been tested before whether species traits can also drive interspecific variation in the contribution of solar radiation to the decomposition process. Here we hypothesize that different litter qualities, through the “afterlife” effects of functional traits^6^,^7^,^21^, also result in species-specific variation in the contribution of solar radiation to leaf litter decomposition. Such trait “afterlife” effects on the contribution of solar radiation are likely because the resorption of nutrients by the plant is incomplete^22^,^23^ and that of recalcitrant compounds such as lignin and tannins probably negligible^6^. Given the unique contribution of solar radiation to the decomposition processes in arid and semi-arid ecosystems, the traits that predict the contribution of solar radiation may be different from the traits that affect the microbially-driven decomposition process which proceeds under shade.

We designed a one-year litter decomposition experiment featuring a factorial design of solar radiation and moisture treatments which together might reveal the interspecific variation in the contribution of solar radiation to leaf litter decomposition among 13 species from Northern China; and we relate such variation to plant functional traits. The aims of our study are (1) to test whether, and by how much, the contribution of solar radiation to leaf litter decomposition rate varies among plant species; (2) to correlate the interspecific variation in the contribution of solar radiation to the variation of the decomposition rate under shade, i.e. mostly microbially-driven decomposition rate; and (3) to attribute such interspecific variation in the contribution of solar radiation to plant functional traits. Specifically, since solar radiation acts directly on the leaf surface, we hypothesize that the leaf surface area per unit mass, i.e. specific leaf area, should scale with the contribution of solar radiation to leaf litter decomposition across multiple species^9^,^24^,^25^.

## Results

**Figure 1:**
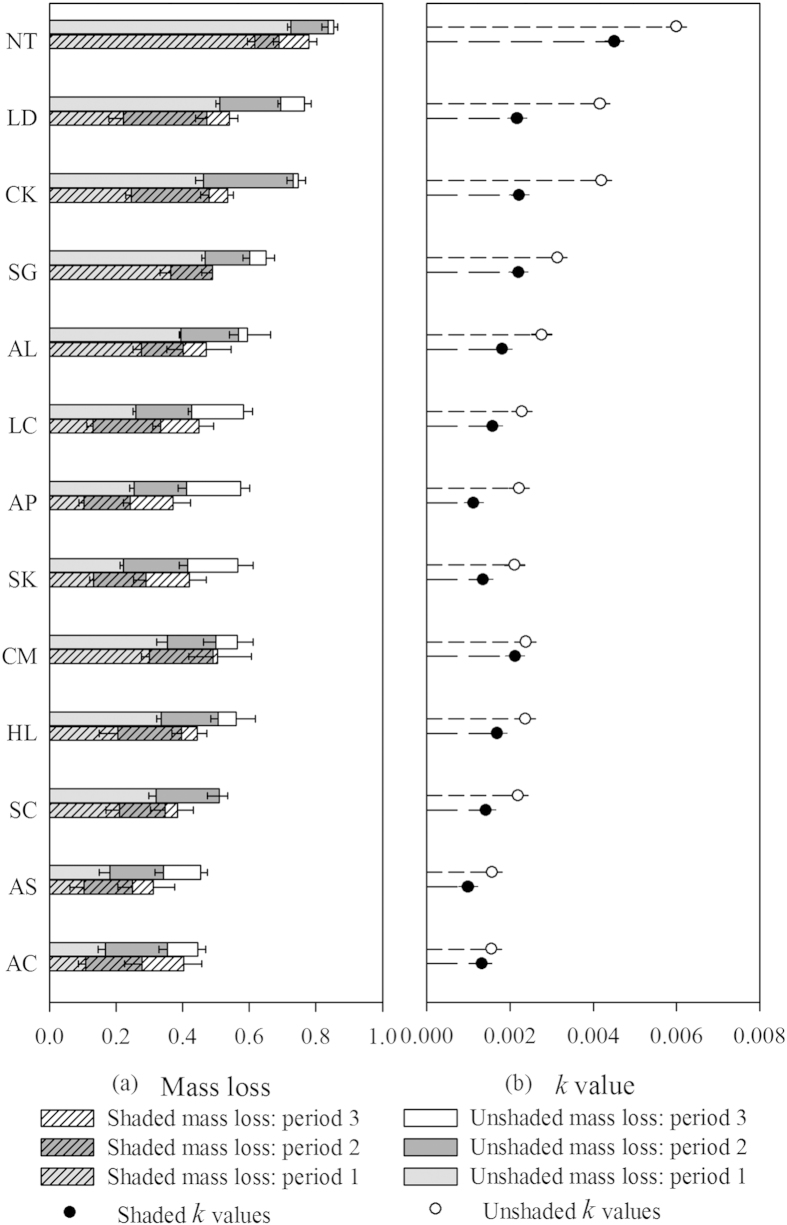
Summary of leaf litter decomposition rates among 13 arid-zone species: (**a**) the mass loss dynamics of 13 species during three harvests (6 months, 9 months, 12 months); (**b**) the decomposition constant *k*-value (*k*_1_, *k*_2_) of 13 species. The empty circles stand for leaf litter decomposition rates under unshaded conditions (*k*_1_); the solid circles for those under shaded conditions (*k*_2_). Period 1-3 represent the duration between three harvests: 0–6 months, 6–9 months and 9–12 months. The Y-axis stands for the 13 species, abbreviated as: NT—*Nitraria tangutorum*, LD—*Lespedeza davurica,* CK—*Caragana korshinskii*, SG—*Salix gordejevii*, AL—*Alhagi sparsifolia*, LC—*Leymus chinensis*, AP—*Agriophyllum pungens*, SK—*Stipa krylovii*, CM—*Calligonum mongolicum*, HL—*Hedysarum laeve*, SC—*Salix cheilophila*, AS—*Achnatherum sibiricum*, AC—*Agropyron cristatum*. Each error bar represents one standard error (se).

The decomposition rates under unshaded (*k*_1_) and shaded (*k*_2_) conditions varied significantly among plant species (Table 1, *k*_1_, *F* = 130, *df* = 12, *P* < 0.001; *k*_2_, *F* = 45.7, *df* = 12, *P* < 0.001). The range of *k*_1_ was from 0.0016 (*Agropyron cristatum*) to 0.0060 (*Nitraria tangutorum*), while the range of *k*_2_ was from 0.0010 (*Achnatherum sibiricum*) to 0.0045 (*N. tangutorum*). Moreover, the difference between *k*_1_ and *k*_2_, i.e. the contribution of solar radiation, also varied significantly among plant species (Table 1, *k*_1_–*k*_2_, *F* = 12.6, *df* = 12, *P* < 0.001). The largest contribution was seen in *Lespedeza davurica* and the smallest contribution in *A. cristatum* (Fig. 1). Note that the interspecific variation in the decomposition rate under shaded conditions was smaller than that of the contribution of solar radiation to leaf litter decomposition rate based on the coefficients of variation (CV, the CV of *k*_1_, *k*_2_ and *k*_1_–*k*_2_ are 0.44, 0.51 and 0.73).

**Figure 2:**
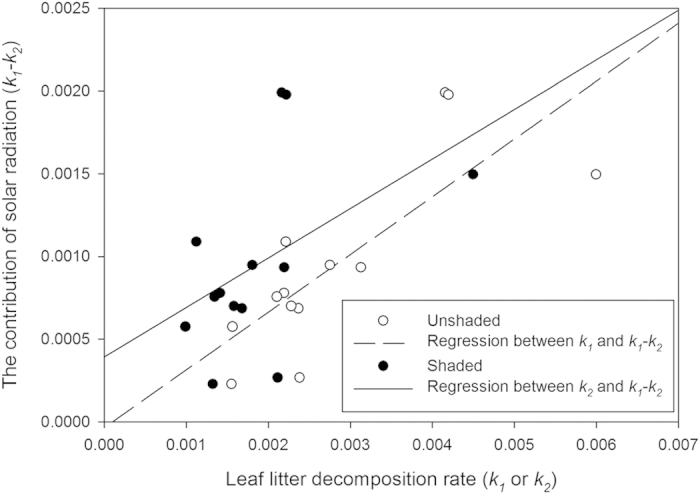
Relationship between the contribution of solar radiation to leaf litter decomposition (*k*_*1*_–*k*_*2*_) and leaf litter decomposition rates: with solar radiation (empty circle) and under shade (solid circle). Regression line was drawn where the correlation was significant (*P* < 0.05). The statistics for these relationships are as follows: with solar radiation: *N* = 13, r = 0.78, *P* < 0.01; under shade: *N* = 13, r = 0.47, *P* = 0.10.

Solar radiation significantly accelerated the decomposition rates (Fig. 1, One-sample T-test, for *k*_1_–*k*_2_ of all 13 species, *t* = 15.6, *P* < 0.001; for each individual species, *k*_1_–*k*_2_ was always greater than 0, *P* < 0.01). Moreover, substrate moisture only had a significant effect on *k*_2_ (Table 1, *F* = 6.15, *df* = 12, *P* = 0.015). There was a significant positive relationship between *k*_*1*_ and *k*_*1*_–*k*_*2*_ (Fig. 2, N = 13, *r* = 0.78, *P* = 0.02) and a marginally significant relationship between *k*_*2*_ and *k*_*1*_–*k*_*2*_ (Fig. 2, N = 13, *r* = 0.47, *P* = 0.10). Significantly different mass losses among species between unshaded and shaded conditions were only detected during period 1 (Fig. 1: 0–6 months, the difference of mass losses for 13 species was significantly greater than 0 during period 1, *t* = 7.05, *P* < 0.001). The species*-*specific pattern for mass loss over time can be seen in Supplementary Fig. S1.

**Figure 3:**
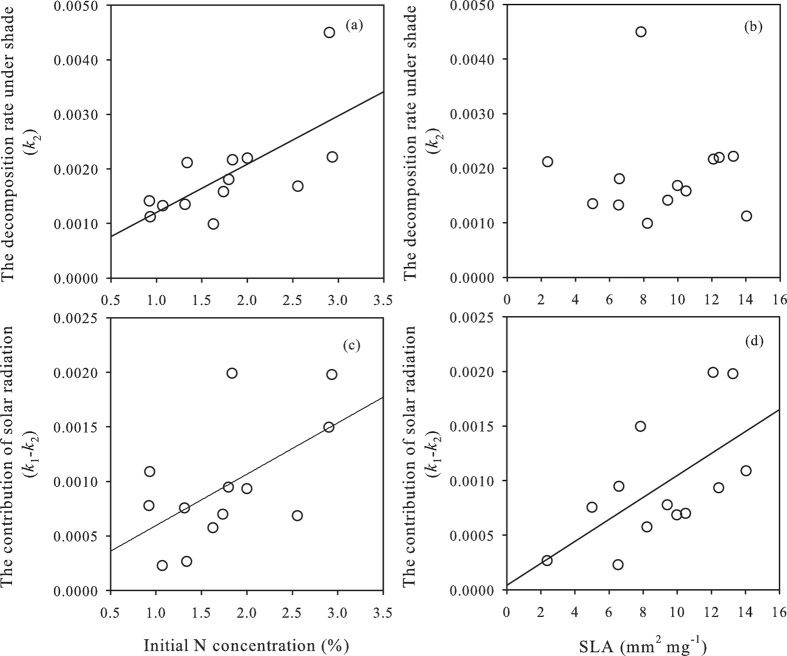
Relationships between functional traits (N, SLA) and *k* values of the decomposition rate under shade (*k*_2_) and the contribution of solar radiation to leaf litter decomposition (*k*_1_–*k*_2_). The statistics for these relationships are as follows: (**a**) *N* = 13, r = 0.68, *P* = 0.01; (b) *N* = 13, r = −0.04, *P* = 0.90; (**c**) *N* = 13, r = 0.57, *P* = 0.04; (**d**) *N* = 13, r = 0.62, *P* = 0.03. Note that significance of these relationships remained the same when removing the outlier in the Fig. 3a,b.

As to the traits expected to influence *k*, total N was positively correlated with *k*_1_, *k*_2_ and *k*_1_–*k*_2_; carbon related traits (total C or C/N) were only negatively correlated with *k*_1_ and *k*_2_, but not significantly related to *k*_1_–*k*_2_; SLA was positively correlated with *k*_1_–*k*_2_, but not significantly related to either *k*_1_ or *k*_2_ (Fig. 3, Supplementary Table S2). Note that data of species traits can be seen in Supplementary Table S3.

## Discussion

Our results provide the first comprehensive empirical evidence for how the effects of solar radiation on leaf litter decomposition vary among plant species, with paramount implications for the role of vegetation composition in carbon release from the extensive sunny parts of the Earth’s land surface. Below we discuss the building blocks and caveats of these findings before allowing ourselves to reinforce this conclusion.

### Interspecific variation in the contribution of solar radiation to leaf litter decomposition rates

Solar radiation markedly increased the leaf litter decomposition rate of (semi-)arid-zone species, leading to 12%–96% increase across our species (Fig. 1, (*k_1_–k_2_*)*/k_2_*). This large range in % increase highlights that the contribution of solar radiation to leaf litter decomposition rates varied significantly according to species-specific traits. The (marginally) positive correlation between *k*_*2*_ and *k*_*1*_–*k*_*2*_ indicates that solar radiation might contribute more to the litter species that already decompose quickly under shade. One possible mechanism is that photodegradation induced by solar radiation might interact with microbial decomposition and thereby contribute more to the species litter decomposition. Moreover, the significant positive correlation between *k*_*1*_ and *k*_*1*_–*k*_*2*_ across species indicates the importance of solar radiation in controlling the leaf litter decomposition rate across species. These results agree with the fast-growing evidence that photodegradation may be particularly important in seasonally dry^26^ or generally dry and less-shady ecosystems^8^,^27^,^28^. These findings are also in line with other single-species studies or litter-mixture studies on solar radiation or photodegradation on decomposition^16^,^24^,^25^,^29^, underlying the facilitation effect of solar radiation on litter decomposition rate across a wide range of species. However, our results did not show the predominant role of solar radiation in determining leaf litter decomposition rates as other research suggested^8^. In our case, a large proportion of mass losses was measured under shaded conditions. This may be partly because most of the yearly precipitation in our study region falls in summer rather than winter, and the combination of high precipitation and high temperature will promote the activities of microbes, increase the relative contribution of microbes to leaf litter decomposition and, thereby, decrease the relative contribution of solar radiation. Additionally, our litterbag method may have also resulted in somewhat reduced effects of solar radiation compared to open-top litter trays^8^. Therefore, our results should be seen as a lower bound on the contribution of solar radiation to decomposition in this system.

In addition, we observed larger interspecific variation in the contribution of solar radiation to leaf litter decomposition rate than that in the microbially-driven decomposition rate. However, we did not notice obvious consequences of such large interspecific variation on the species’ litter abundance on the soil surface in arid ecosystems. One reason is that, in arid and semi-arid regions, a large proportion of litter remains in standing dead matter of herbs. This results in little leaf litter of different species on the ground during the year, except for the beginning of the senescence season. Another reason may be that litter production in arid and semi-arid regions is much less than that in forests or other humid ecosystems, and that leaf size is so small in arid regions that incompletely decomposed litter may get mixed with or covered by the soil. The effects of soil-litter mixing might partly mitigate the large interspecific variation in the contribution of solar radiation to leaf litter decomposition rates^30^. A third possible reason could theoretically be that in arid and semi-arid regions interspecific decomposition rates were negatively correlated between solar radiation-induced decomposition and microbially-driven decomposition under shaded conditions; However we observed the opposite (Fig. 2).

### Traits as drivers of the variation in the contribution of solar radiation to leaf litter decomposition rate

The contribution of solar radiation across species (*k*_*1*_–*k*_*2*_) correlated positively with SLA (Fig. 3, *P* = 0.005, r = 0.731). Our result is consistent with intraspecific evidence of three species—from the genera *Populus*, *Pinus* and *Juniperus* respectively—for which the contribution of solar radiation was proportional to exposed litter surface area per unit dry mass^24^,^25^. One explanation may be that high SLA provides an extensive surface for gas exchange with the atmosphere and exposure to sunlight thereby facilitates photodegradation^31^,^32^,^33^. Here, a caveat is that for standing dead litter that remains on the plant, leaf angles may influence the decomposition rates *in situ*. Self-shading and litter shading may play a role too, although this role is likely small in most dry, less-shady ecosystems with very open and interrupted canopies. On the other hand, our results did not show a significant relationship between SLA and leaf litter decomposition rate under shade, suggesting that other traits or abiotic decomposition processes might contribute to the mass loss, such as freeze-thaw action^34^. However, this result is opposite to some previous studies which indicated that species litters with less structural tissue or high-quality carbon, higher SLA and nutrient content, and lower secondary compounds, normally decompose faster^7^,^35^,^36^. Actually, in some cases, the significant relationship between SLA and leaf litter decomposition rates was only detected when other leaf traits were considered at the same time^36^.

The contribution of solar radiation across species also correlated positively with N concentration of initial litter, indicating such contribution may also be due to the interaction between solar radiation and microbes. The N concentration has often been related to leaf litter decomposition rates because initial litter N might exert an influence on the formation of recalcitrant complexes of N-rich proteins with secondary metabolism compounds^37^ or on the abundance and activity of decomposers, especially microbes^38^. Higher N concentration of initial litter may increase the microbial activity, leading to more efficient microbes using the by-products of solar radiation via photodegradation and thereby increase the leaf litter decomposition rate. In addition, solar radiation can also increase the temperature and in turn increase the microbial activity, leading to higher decomposition rate. In our study, we considered that the contribution of solar radiation might be partly due to its positive effect on abiotic factors such as temperature. These results might be indirect evidence of the positive effect of solar radiation on microbes and thereby on the decomposition of leaf litter. However, solar radiation, in some cases, can also lead to heat stress or desiccation of decomposers and thereby lead to decreased litter decomposition rates^39^,^40^. We cannot rule out this possibility based on our results, because we only quantified the net effect of solar radiation on leaf litter decomposition rates among species. We suggest that in future studies the positive and negative effects of solar radiation on decomposers and decomposition should be differentiated and quantified.

Lignin might play a dual role in litter decomposition in terrestrial ecosystems^41^. Lignin is typically regarded as a recalcitrant material and thereby could limit microbially driven decomposition; on the other hand, it will promote solar radiation-driven mass loss due to the absorption spectrum of lignin^42^. Thus, the same trait may have distinct consequences for the decomposition rates in the presence or the absence of solar radiation. Therefore, the microbially-driven decomposition is possibly decoupled with the contribution of solar radiation to leaf litter decomposition owing to different litter traits determining each process. Regrettably, we did not measure the lignin concentration of our initial and decomposed litter to test this possible dual role of lignin on a species level. However, one of the leaf traits we studies, SLA, tends to scale well (and negatively) with lignin concentrations across species^42^,^43^, and SLA was also only correlated with the contribution of solar radiation to decomposition but not with the decomposition rate under shade (Fig. 3).

For dry and sunny ecosystems, strong solar radiation and moisture deficit may exert a particular influence on leaf surface traits. In general, the problem of excess photons can be addressed by constructing more photosynthetic capacity. However, there may be an upper limit to this strategy, and many species show additional epidermal traits including waxes and trichomes that restrict the passage of some UV into the leaf^44^. The effects of these traits on the contribution of solar radiation to litter decomposition are not well constrained: they likely depend on the longevity of the epidermal structures and properties after leaf death, which is poorly understood. We propose leaf surface characters as promising traits to evaluate the contribution of solar radiation to litter decomposition, which may be sufficiently important for integration into global circulation models of carbon exchange between the terrestrial biosphere and atmosphere.

Our results provide the first comprehensive empirical evidence of species-based differences in the contribution of solar radiation to decomposition processes. The large interspecific variation in such contribution tended to be positively correlated with that in the decomposition rates under shade, and could be attributed to the variation in SLA among species. Moreover, we suggest that leaf surface traits should be integrated into future studies focusing on the trait-decomposition relationship in semi-arid and arid regions with strong solar radiation. Altogether, our findings demonstrate the importance of vegetation composition for the contribution of solar radiation to carbon release from the earth surface in dry, less-shady environments, which should have implications for the global carbon balance.

## Materials and Methods

### Study site

The experiment was conducted at the Ordos Sandland Ecological Station (OSES; 39º29′37.6′′ N, 110º11′29.4′′ E, 1300 m a.s.l.; Institute of Botany, the Chinese Academy of Sciences) located in the Mu Us Sandland in Inner Mongolia, China. Long-term mean annual precipitation is 260–450 mm, 60–70% of the precipitation falling between July and September^45^. The mean annual temperature is 7.5–9.0 °C with a mean maximum of 20–24 °C in July and a mean minimum of −8 to −12 °C in January. Soils have developed in aeolian sand, having a complete C horizon and sometimes a thin A horizon. The dominant vegetation consists of (semi-)shrubs such as *Artemisia ordosica* Krasch., *Hedysarum leave* Maxim., *Salix psammophila* Z. Wang & Chang Y. Yang and *Sabina vulgaris* Antoine^46^. Mean annual solar irradiance is 2800–3100 hr, and annual total solar radiation is 138–150 kcal cm^−2^.

### Litter decomposition experiment

We collected newly senesced leaves (hereafter called “litter”) at the end of the growing season mainly from four locations (Fukang, Ordos, Xilingol, Naiman) along a precipitation gradient ranging from 160 mm y^−1^ to 370 mm y^−1^ in the arid and semi-arid northwest of China (for details^47^). From each site, litter of one to four dominant or abundant species was chosen as litter materials used in our experiment. In total, 13 species were selected at four sites (details in Supplementary Table S3). The litter was all stored in OSES, air-dry and in the dark for more than 10 days.

We used 6 cm × 6 cm white litter bags made of net curtain material with a mesh size of 0.5 mm, suited for the small leaf sizes of some xeric plant species. This mesh intercepted a small fraction (<10%) of the light, not enough to substantially diminish solar radiation of the litter. Each litterbag was filled with 0.5 ± 0.1 g litter forming a monolayer to ensure minimal self-shading. Before filling litterbags with each species, a subsample was weighed, then oven dried at 75 °C for 48 hr, and reweighed. Initial dry mass for each litter sample was calculated from the moisture content of this subsample. We also measured leaf (litter) traits that were considered potential predictors of litter decomposition rates. Five sub-samples for each species’ initial litter were analyzed for total C by standard wet combustion and for total N concentration by the semi-micro Kjeldahl method. Data for specific leaf area (SLA; leaf area per dry mass) of green leaves of the same species had been collected in 2008. Note that these SLA data were measured based on green leaves which were different from the leaf litter used in this study.

We utilized a construction with ten open-top cement boxes, built in 1995, as the litter bed for our study, each box measuring 2 m (length) × 1.5 (width) m × 1.2 m (depth) and filled with 3 m^3^ sandy soil. We replaced the top 30 cm of soil with homogeneous soil (sandy soil collected from the surroundings near OSES) for creating a consistent incubation environment. The soil surface was leveled and kept 15–20 cm lower than the top of the box in order to avoid the litterbags being taken away by the strong wind in the study region.

The factorial design included manipulations of solar radiation and substrate moisture. To test for the effect of solar radiation, two layers of shading mesh were attached onto the top of each individual litterbag to block out most (>95%) of the solar radiation. Moreover, in order to detect the treatment effect on other physical factors, notably the temperature, we put six button thermometers into six empty litterbags respectively at the end of October: three under unshaded litter bags and the other three under shaded litter bags. There was a slight decrease of daily mean temperature due to shading (Supplementary Fig. S4). This may have affected microbial respiration, which is temperature-sensitive. We considered that the contribution of solar radiation to leaf litter decomposition may be partly due to the higher temperature under solar radiation. The substrate moisture treatment entailed adding water once a week. The amount of added water was proportional to seasonal precipitation, and we added 100 mm in total: more specifically, we added 17 mm water in spring, 65 mm in summer and 18 mm in autumn, but in winter we did not add any water owing to ambient frost conditions. The total amount of water added to each box during each season was 0.051 m^3^ in spring, 0.195 m^3^ in summer and 0.054 m^3^ in autumn (the size of the box was 3 m^2^). Therefore, in spring we added around 0.004 m^3^ every week; in summer around 0.016 m^3^ and in autumn around 0.005 m^3^. This increase in yearly precipitation would represent a change from semi-arid to semi-humid climate. We standardized the speed of water flow (around 4 L min^−1^) and the time of spraying. The water was sprayed directly onto the litter bags.

On 22 Dec. 2009, all litterbags were randomly placed flat on the sand surface, without overlap and leaving 30 cm buffer zones in the south and 15 cm buffer zones on the other sides to prevent shading effects of the boxes, which would to some extent have decreased the effect of shading on the soil temperature. For each treatment there were 5 replications. Litterbag harvests were on 22 Mar., 22 Jun., 22 Sep. and 22 Dec. 2010, respectively. Contaminants in the remaining litter were removed by hand and the remaining litter was reweighed after oven-drying at 75 °C for 48 hr. For the harvested litter, we only measured the oven-dry mass of the remaining litter for each litter bag. Note that we only used the data of the last three harvests in our analyses afterwards due to the lack of moisture treatment during winter period.

### Data analysis

The decomposition constant *k*-values (hereafter called *k* in short) were first calculated for each treatment combination and its replicates based on the mass losses of three harvests^48^. For each *k*, the mass loss was first changed to the mass remaining; the mass remaining was then *ln*-transformed, followed by regression of the *ln*-transformed percent mass remaining against time. The slope of the regression line is the exponential decay constant, i.e. *k* (d^−1^).

In order to examine the contribution of solar radiation to leaf litter decomposition rates, we further defined *k*_1_ as the decomposition rate under unshaded conditions, which approximately stands for the ambient decomposition rate including microbially-driven decomposition, the contribution of solar radiation and their interaction (presumably slightly reduced due to the shading by litter bag mesh); *k*_2_ as the decomposition rate under shaded conditions, which approximately stands for the microbially-driven decomposition rate. Hence, *k*_1_–*k*_2_ stands for the difference of decomposition rates between unshaded and shaded conditions, interpreted as the contribution of solar radiation to leaf litter decomposition rate in our study.

**Figure 4:**
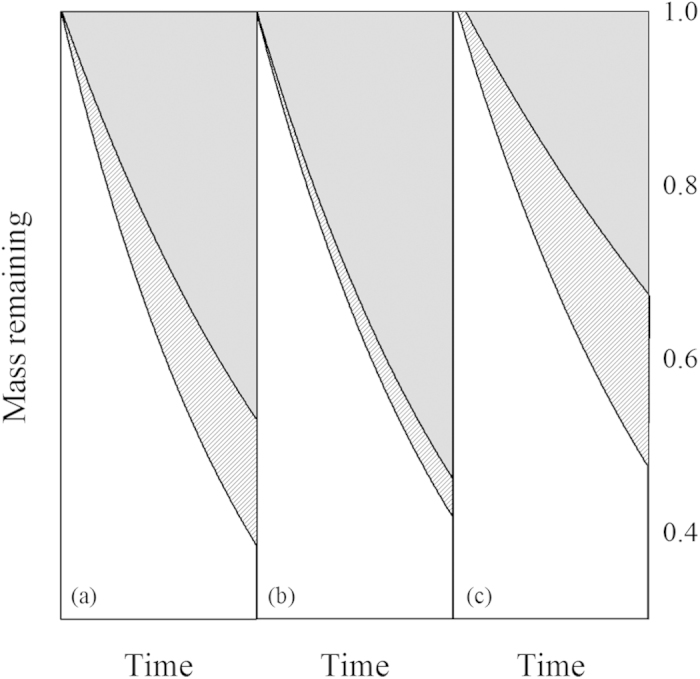
Explanation of the contribution of solar radiation to the decomposition constant *k* and mass loss over time. Fig. 1a is drawn based on the average mass losses of all 13 species in our study under unshaded and shaded conditions; exponential models were selected to fit the mass remaining during decomposition; Fig. 4b,c show possible effects of solar radiation (*Calligonum mongolicum* and *Agriophyllum pungens* respectively) on the decomposition constant *k* and mass loss over time. The slope of tangent lines stands for the *k*. The light grey areas stand for the decomposition under shaded conditions which was induced mostly by microbial decomposition; the forward diagonal shaded areas stand for the decomposition under unshaded conditions which was induced by solar radiation, such as visible light and UV radiation, directly and indirectly through interactions with microbes and other factors. Note that the moisture treatments were not considered here and therefore the mass loss data under different moisture levels were treated as more replicates under different solar radiation treatments.

We tested the normality of our data before all the analyses. Then, we used the general linear model (SPSS 15.0) to test the effects of species and moisture on the leaf litter decomposition rate (*k*_1_, *k*_2_ and *k*_1_–*k*_2_) across all 13 species. Moreover, we calculated the coefficients of variation for *k*_1_, *k*_2_ and *k*_1_–*k*_2_ to evaluate the degree of interspecific variation of decomposition rate under unshaded and shaded conditions. Figure 4 gives a detailed example of possible interspecific variation of the contribution of solar radiation to leaf litter decomposition. We also calculated the mass losses for each solar radiation treatment as the average between two moisture levels and examined the difference of mass losses under unshaded and shaded conditions throughout the three harvests. One sample T-test was used to examine the effect of solar radiation on *k* values and mass losses, and simple regression was used to examine the relationships among *k*_*1*_, *k*_*2*_ and *k*_*1*_–*k*_*2*_. In the end, we used both Pearson’s correlation analysis and general linear regression analysis to test the relationships between traits (including litter traits: total C, total N and C/N and SLA of the green leaves) and the *k*-values (Supplementary Table S5). Again, we verified residual distribution graphically using predicted/residual plots and normal probability plots.

## Additional Information

**How to cite this article**: Pan, X. *et al.* Functional traits drive the contribution of solar radiation to leaf litter decomposition among multiple arid-zone species. *Sci. Rep.* **5**, 13217; doi: 10.1038/srep13217 (2015).

## Supplementary Material

Supplementary Information

## Figures and Tables

**Table 1:** Summary of results of linear regression model.

Dependent variable	Independent variable	*df*	*F*	*P*
*k*_1_	Species	12	**130.0**	**<0.001**
	Substrate moisture	1	1.3	0.263
*k*_2_	Species	12	**45.7**	**<0.001**
	Substrate moisture	1	**6.2**	**0.015**
*k*_1_–*k*_2_	Species	12	**12.6**	**<0.001**
	Substrate moisture	1	2.6	0.107

The dependent variables are the decomposition constant *k*-values and combinations thereof, where *k*_1_ stands for the decomposition rate at full solar radiation and *k*_2_ stands for the decomposition rate under shade. The independent variables are species identity and substrate moisture treatments (adding water or not). Significance is shown in bold (*P* < 0.05).
