# Supplementary material for: Functional traits drive the contribution of solar radiation to leaf litter decomposition among multiple arid-zone species
Source: Sci Rep. 2015 Aug 18;5:13217. doi: 10.1038/srep13217 (PMC4539537; doi:10.1038/srep13217)
Supplement: Supplementary Information [file srep13217-s1.pdf]

Functional traits drive the contribution of solar radiation to leaf litter decomposition among multiple arid-zone species

Xu Pan[1,2,3], Yao-Bin Song[1], Guo-Fang Liu[3], Yu-Kun Hu[3], Xue-Hua Ye[3],William K. Cornwell[4,5], Andreas Prinzing[6], Ming Dong[1,3*], Johannes H.C. Cornelissen[4]

[1]Key Laboratory of Hangzhou City for Ecosystem Protection and Restoration, College of Life and Environmental Sciences, Hangzhou Normal University, Hangzhou, China

[2]Institute of Wetland Research, Chinese Academy of Forestry, Beijing, China

[3]State Key Laboratory of Vegetation and Environmental Change, Institute of Botany, Chinese Academy of Sciences, Beijing, China

[4]Department of Ecological Science, VU University, Amsterdam, the Netherlands

[5]Evolution & Ecology Research Centre, School of Biological, Earth and Environmental Sciences, University of New South Wales, Sydney, Australia

[6]Université de Rennes 1, Centre National de la Recherche Scientifique, Rennes, France

Supplementary Fig. S1 The pattern for mass losses over time across 13 arid-zone species.

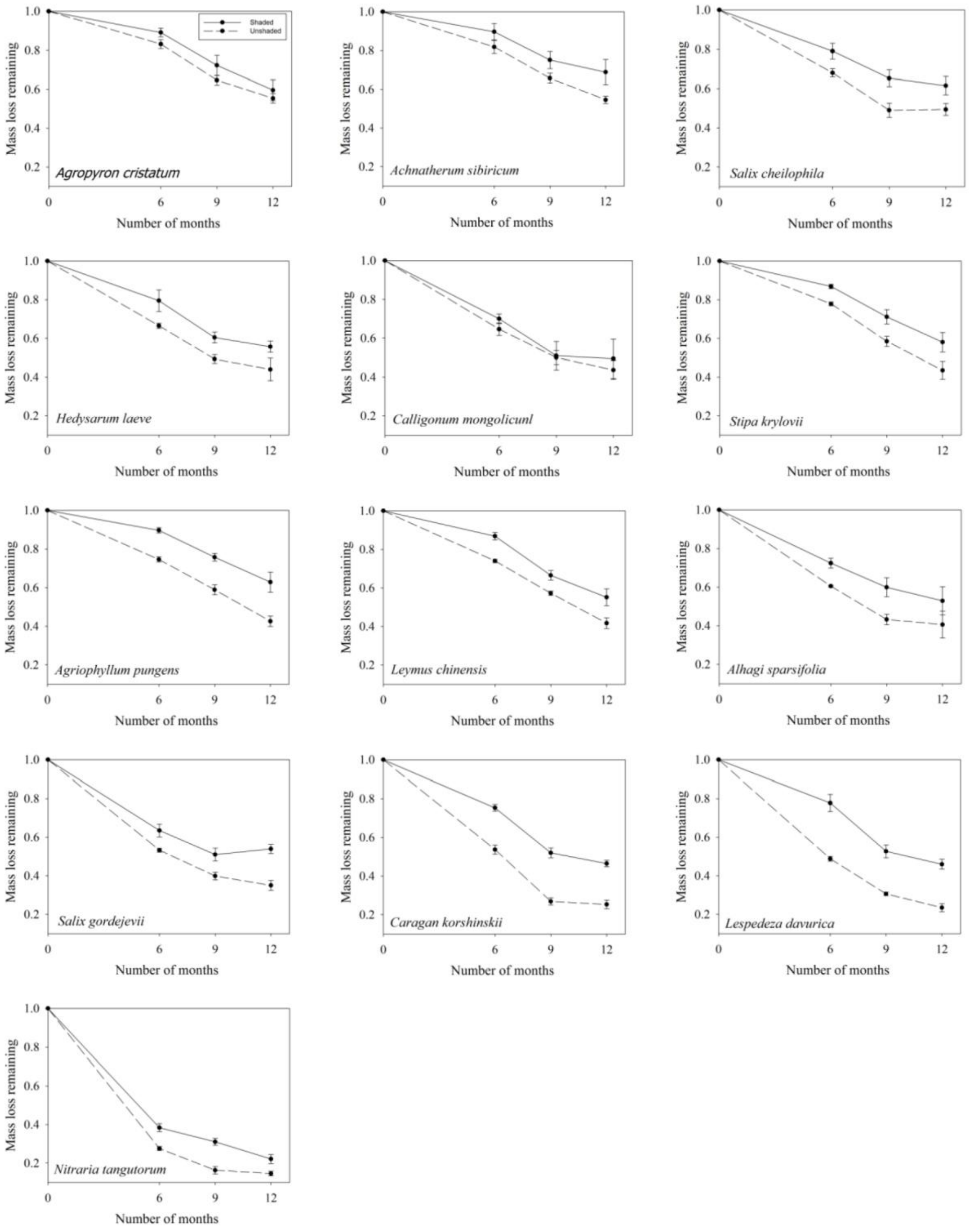

Supplementary Table S2 General linear regression between the decomposition constant *k*-values (k_1, k_2, k_1- k_2) and the litter traits (total C, total N, C/N, SLA). A best subset search was applied to avoid multicollinearity among independent variables (adjusted R^2 criterion optimizing R^2 while accounting for numbers of variables, STATISTICA v. 7.0).

For k_1:

	Comment	Parameter	Std Err	*t*	*P*
Intercept		0.0018	0.0026	0.69	0.51
Total N		0.0022	0.0008	2.64	**0.03**
Total C		*-0.0001*	*0.0000*	*-2.14*	*0.06*
C/N		0.0001	0.0000	1.26	0.24
SLA	Pooled				

For k_2:

	Comment	Parameter	Std Err	*t*	*P*
Intercept		0.0026	0.0017	1.53	0.16
Total N		0.0014	0.0006	2.62	**0.03**
Total C		-0.0001	0.0000	-3.11	**0.01**
C/N		0.0000	0.0000	1.31	0.22
SLA	Pooled				

For k_1-k_2:

	Comment	Parameter	Std Err	*t*	*P*
Intercept		-0.0004	0.0004	-1.08	0.31
Total N		*0.0004*	*0.0002*	*2.06*	*0.07*
Total C	Pooled				
C/N	Pooled				
SLA		0.000082	0.000035	2.32771	**0.04**

Supplementary Table S3 Plant species used in the decomposition experiment. N or F stands for Nitrogen fixer (NF) or not (/); SFG stands for Shrub (S), Forb (F) or Grass (G); C stands for total carbon concentration of initial litters; N stands for total nitrogen concentration of initial litters; Site 1-5 stands for Ordos, Xilingol, Naiman and Fukang.

Species list	Site	NF	SFG	C (%)	N (%)	C/N	SLA (g cm^{-2})
Hedysarum leave Maxim.(HL)	1	NF	S	50.19	2.56	19.64	9.98
Lespedeza davurica (Laxm.) Schindl. (LD)	1	NF	S	48.91	1.84	26.64	12.09
Agriophyllum pungens Link ex A.Dietr. (AP)	1	/	F	43.70	0.93	46.88	14.03
Salix cheilophila C.K.Schneid. (SC)	1	/	S	54.66	0.92	59.28	9.41
Leymus chinensis (Trin.) Tzvelev. (LC)	2	/	G	50.22	1.74	28.93	10.49
Achnatherum sibiricum (L.) Keng. (AS)	2	/	G	49.58	1.63	30.50	8.22
Stipa krylovii Roshev. (SK)	2	/	G	51.36	1.31	39.08	5.00
Agropyron cristatum (L.) Gaertn. (AC)	2	/	G	52.38	1.07	48.96	6.52
Caragan korshinskii Kom. (CK)	3	NF	S	52.20	2.93	17.79	13.26
Salix gordejevii Y.L.Chang & Skvortsov. (SG)	3	/	S	54.75	2.00	27.37	12.43
Calligonum mongolicum Turcz. (CM)	4	/	S	37.20	1.34	27.81	2.36
Alhagi sparsifolia Shap. (AL)	4	NF	S	47.42	1.80	26.41	6.57
Nitraria tangutorum Bobrov. (NT)	4	/	S	35.43	2.90	12.21	7.84

Nomenclature of species follows

Song *et al.*, 2002; Zeng *et al.*, 2002; Liu *et al.*, 2007; Zhang & Zhu, 2009 and Flora of China.

Liu F.H., *et al*. Responses of *Hedysarum laeve*, a guerrilla clonal semi-shrub in the Mu Us Sandland, to local sand burial. *Front Biol Chin* **2**, 431-436 (2007).

Song M.H., Dong M., Jiang G.M. Importance of clonal plants and plant species diversity in the Northeast China Transect. *Ecol Res* **17**, 705-716 (2002).

Zeng F.J., *et al*. Water relation characteristics of *Alhagi sparsifolia* and consequences for a sustainable management. *Sci Chin Ser D: Earth Sci* **45**, 125-131 (2002).

Zhang Q., Zhu X.T. Microsatellite DNA loci from the drought desert plant *Calligonum mongolicum*; Turcz. (Polygonaceae). *Conserv Genet* **10**, 1891-1893 (2009).

Supplementary Fig. S4 Daily mean temperature under unshaded and shaded litter bags from 26th October to 22nd December. Solid line represents the temperature under unshaded conditions; dashed line represents the temperature under shaded conditions.

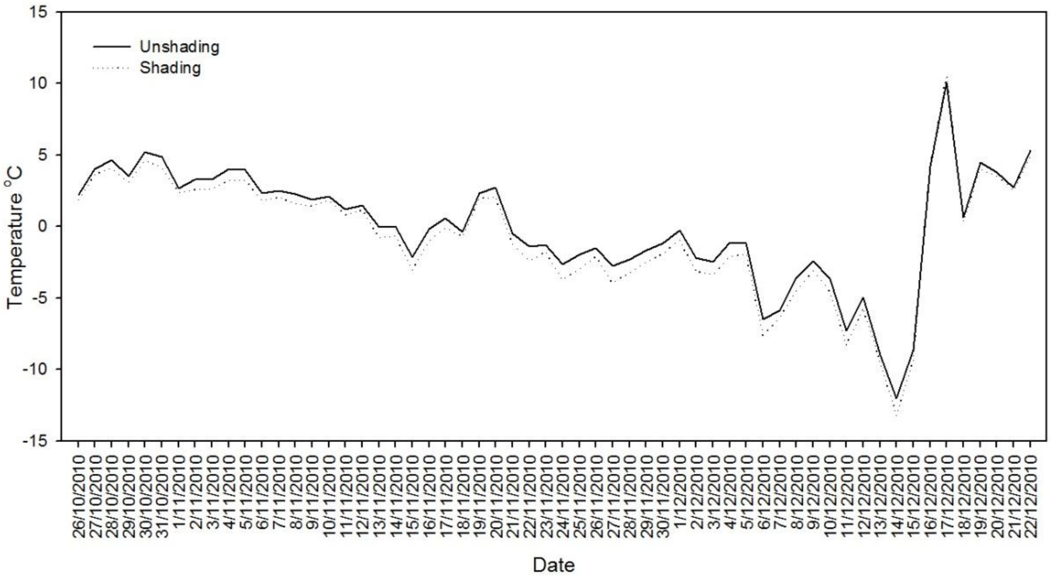

Supplementary Table S5 Pearson's correlation coefficient between the decomposition constant *k*-values (k_1, k_2, k_1- k_2) and the litter traits (total C, total N, C/N, SLA). Note k_1 stands for the decomposition rate under unshaded conditions; k_2 stands for the decomposition rate under shaded conditions.

Traits	Total C		Total N		C/N		SLA	
k-values	R	*P*	R	*P*	R	*P*	R	*P*
k_1	-0.434	0.138	**0.738**	**0.004**	**-0.653**	**0.016**	0.247	0.416
k_2	**-0.588**	**0.035**	**0.681**	**0.010**	**-0.646**	**0.017**	-0.040	0.898
k_1- k_2	-0.040	0.897	**0.574**	**0.040**	-0.438	0.134	**0.616**	**0.025**
